# Inflammatory Stimuli Significantly Change the miRNA Profile of Human Adipose-Derived Stem Cells

**DOI:** 10.1155/2018/1340341

**Published:** 2018-01-28

**Authors:** Zicheng Wang, Haili Li, Li Cheng, Zhongyuan Zhang, Hao Wang, Tongde Lv, Jian Lin, Liqun Zhou

**Affiliations:** ^1^Department of Urology, Peking University First Hospital, Beijing 100034, China; ^2^Institute of Urology, Peking University, Beijing 100034, China; ^3^Department of Ophthalmology, Peking University First Hospital, Beijing 100034, China; ^4^National Urological Cancer Center, Beijing 100034, China; ^5^Department of Obstetrics and Gynaecology, Peking University First Hospital, Beijing 100034, China

## Abstract

**Background:**

Human adipose-derived stem cells (hASCs) have been shown to have immunoregulatory properties in many studies. However, the mechanisms remain unknown. miRNAs are associated with many cellular processes, including immune responses. Thus, we hypothesized that miRNAs act as immunoregulators when hASCs are stimulated by inflammatory environments.

**Methods:**

A set of cytokines was used to stimulate the hASCs in the cytokine group, while no cytokines were used to stimulate the cells in the normal group. A microarray was used to obtain the miRNA expression profile of hASCs, and RT-PCR was used to validate the miRNAs that were differentially expressed between the two groups. Target genes were predicted using online databases, and KEGG analysis was performed to identify the pathways enriched by the target genes of all the differentially expressed miRNAs.

**Results:**

Five miRNAs were significantly upregulated, and 2 miRNAs were downregulated in the cytokine group compared with the normal group. We identified several immune-related pathways that are targeted individually or collectively by those miRNAs.

**Conclusion:**

Inflammatory stimuli changed the miRNA expression profile of hASCs. miRNAs may play a pivotal role in the immune response in hASCs and may be targets through which the immunoregulatory functions of hASCs can be enhanced.

## 1. Introduction

Mesenchymal stem cells (MSCs) were originally identified in mouse bone marrow but have since been shown to exist in nearly all tissues. In addition to possessing well-known self-renewal and multipotent differentiation capabilities, MCSs also possess immunomodulatory properties of great clinical value. These properties are responsible for an increasing number of clinical trials in which allogenic MSCs have been used to treat diseases.

Human adipose-derived stem cells (hASCs), which are stem cells within the adipose tissue, have recently been shown to be one of the most promising MSC types. Easily and safely obtainable adipose tissues yield significantly more stem cells than the bone marrow without causing much patient discomfort. In addition to being applied in regeneration medicine, hASCs have also been used to treat autoimmune diseases and graft-versus-host disease (GVHD), based on the immunomodulatory effects they have shown in vivo. In a phase III randomized control study, Cx601 cells, which are expanded allogenic hASCs, were shown to be effective and safe in the treatment of complex perianal fistulae in Crohn's disease [1]. However, the mechanisms by which ASCs exert their therapeutic effects remain unknown.

MicroRNAs (miRNAs) are endogenous non-coding RNAs (~23 nt) that play an important role in the posttranscriptional repression of their target genes by binding to complementary sequences [2]. They are involved in a variety of cellular processes, such as development, proliferation, and apoptosis. Hundreds of expression profiling studies have clarified that tumors exhibit different miRNA expression patterns than normal tissues and that some miRNAs have a dramatic influence on tumorigenesis [3–5]. Furthermore, several miRNAs have been demonstrated to be associated with inflammatory diseases [6] and are required for normal immune function [7].

In this study, we hypothesized that the role of hASCs in inflammatory diseases may be influenced by miRNA expression patterns. Elucidating the miRNA expression profile of hASCs under inflammatory stimulation may help in identifying specific miRNAs that are differentially expressed under inflammatory conditions, thereby facilitating the enhancement of hASC immunoregulatory function. We performed microarray studies and found that 7 miRNAs are differentially expressed between stimulated and unstimulated hASCs. Increases or decreases in the expression of these miRNAs may be involved in the mechanism by which hASCs regulate the immune response and may be used to improve hASC function.

## 2. Methods

### 2.1. Ethics Statement

This study was performed in accordance with the Declaration of Helsinki and was approved by the Peking University First Hospital Institutional Review Board. Human subcutaneous adipose tissues were obtained from patients undergoing elective surgery who gave written consent regarding the use of their tissues for this study.

### 2.2. Generation of hASCs

The adipose tissue samples were processed as described in a previous study [8]. Briefly, the tissues were washed 3 times in sterile phosphate-buffered saline (PBS), mainly to remove red blood cells, and then minced into 1 mm^3^ pieces with eye scissors. An equal volume of 0.1% collagenase type I was used to digest the tissue pieces under horizontal shaking for 1 h at 37°C. The cell pellets were subsequently obtained by centrifugation at 1300 rpm for 3 min and resuspended in DMEM supplemented with 10% fetal bovine serum, after which they were filtered through a 100 *μ*m filter. The hASCs were then seeded in a 10 cm culture dish and incubated in a humidified atmosphere at 37°C with 5% CO_2_. Cells at passage 3 were used for the experiments.

### 2.3. Flow Cytometry

hASCs from passage 3 were harvested by 0.25% trypsin/EDTA and washed in flow cytometry buffer (FCB, 1 × PBS, 1% FBS). Cell aliquots (1 × 10^5^ cells) were stained with fluorescein isothiocyanate- (FITC-) conjugated primary antibodies to CD31, CD44, CD45, CD73, CD90, or CD105 (BioLegend, San Diego, CA, USA) on ice for 30 min. FITC-conjugated mouse IgG1 was used as an isotype control. Flow cytometry was performed using a cell sorter (BD, San Jose, CA, USA), and the data were analyzed using FlowJo software (FlowJo LLC, Ashland, OR, USA).

### 2.4. Adipogenic Differentiation and Oil Red O Staining

Adipogenesis was induced by culturing hASCs in adipogenic medium (AM) for 2 weeks. AM, whose composition has been described previously, comprises the following [9]: DMEM supplemented with 10% FBS, 1 *μ*M dexamethasone, 10 *μ*M insulin, 200 *μ*M indomethacin, and 0.5 mM isobutyl-methylxanthine (IBMX). Differentiation was confirmed using Oil Red O staining. Briefly, the cells were fixed in 4% paraformaldehyde for 1 h at room temperature and washed with 70% ethanol, after which they were incubated in Oil Red O reagent for 3–5 min at room temperature. After several washes with distilled water, the cells were counterstained with hematoxylin.

### 2.5. Osteogenic Differentiation and von Kossa Staining

We used a commercially available osteogenesis differentiation kit (Gibco) as directed by the manufacturer to induce hASC osteogenesis. After incubating in differentiation medium for 3 weeks, the cells were fixed in 4% paraformaldehyde for 1 h at room temperature and washed at least 3 times with distilled water. The cells were then stained with 1 ml of von Kossa reagent (Leagene, Beijing, China) under UV light for 10 min.

### 2.6. hASC Stimulation in Inflammatory Conditions

hASCs were cultured either in normal medium or under inflammatory conditions for analysis. To mimic the inflammatory environment, we treated the cells with combinations of the following cytokines, as previously described [10]: IL-1*β* (25 ng/ml), TNF-*α* (50 ng/ml), IFN-*α* (10 ng/ml), and IFN-*γ* (50 ng/ml) (all from PeproTech, Rocky Hill, NJ, USA). Briefly, the cells were treated with the above cytokines overnight and then collected for miRNA extraction.

### 2.7. RNA Extraction

Total RNA was extracted using a mirVana™ RNA Isolation Kit (Applied Biosystems, AM1556), according to the manufacturer's instructions. RNA concentrations were quantified by NanoDrop ND2000 (Thermo Scientific, MA, USA), and RNA integrity was assessed by agarose gel electrophoresis.

### 2.8. miRNA Array

Affymetrix miRNA 4.0 (Affymetrix, Inc., Santa Clara, CA, USA) was used in this experiment. RNA sample labeling, microarray hybridization, and washing were performed according to the manufacturer's standard protocols. Briefly, a poly A tail was added to total RNA, after which the RNA was biotin-labeled and hybridized onto the microarray. After being washed and stained, the arrays were scanned by an Affymetrix Scanner 3000 (Affymetrix 7G).

### 2.9. Validation of miRNA Expression

We used a stem-loop RT-PCR method to quantify the expression levels of various miRNAs. Reverse transcriptase reaction mixtures containing RT/RI enzyme mix, ES reaction mix, gDNA remover (all from Transgene, AE301, Beijing, China), RNA samples, and stem-loop RT primers were used for these experiments. The reactions were incubated for 30 min at 16°C, 30 min at 42°C, and 5 min at 85°C and then held at 4°C. Real-time PCR was performed using a Transcript® Green miRNA Two-step qPCR SuperMix Kit (Transgene, AQ202, Beijing, China) on an Applied Biosystems 7500 Real-Time PCR System. The 10 *μ*l PCR mixture included 1 *μ*l of RT product, 5 *μ*l of Transcript Tip Green qPCR SuperMix, 0.2 *μ*l of forward primer, and 0.2 *μ*l of reverse primer. The mixtures were incubated in a 96-well plate at 94°C for 30 s, after which they were reacted for 40 cycles of 94°C for 5 s and 60°C for 34 s. All the reactions were run in triplicate. The Ct (threshold cycle) was defined as the cycle number at which the fluorescence passed the threshold. U6 was used as an internal control. The ΔΔCt method, which has been described previously [11], was used to calculate the relative expression levels of the miRNAs.

### 2.10. Statistical and Bioinformatics Analysis

Command Console software (version 4.0, Affymetrix, Santa Clara, CA, USA) was used to analyze the array images to obtain the necessary raw data, and then Expression Console software (version 1.4.1, Affymetrix, Santa Clara, CA, USA) was used to perform RNA normalization. To define the expression profiles of each variant, we performed one-way ANOVA with Transcriptome Analysis Console software (version 3.1, Affymetrix, Santa Clara, CA, USA). miRNAs with a *p* < 0.05 and a |log2(fold change)| > 1 were identified as significantly differentially expressed miRNAs. Hierarchical clustering was performed to show the differences in the expression patterns of these miRNAs among samples.

The target genes of the differentially expressed miRNAs were predicted using the following 5 databases: miRanda, miRDB, miRTarBase, TargetScan, and miRecords. To ensure the accuracy of our prediction, we selected genes enrolled in at least two databases as target genes and subjected them to further analysis.

Finally, KEGG analysis was performed to determine the pathways in which the target genes of these differentially expressed miRNAs are involved. In addition, the target immune function-related pathways of individual miRNAs were predicted using DIANA-miRPath v3.0.

## 3. Results

### 3.1. hASC Characterization

**Figure 1:**
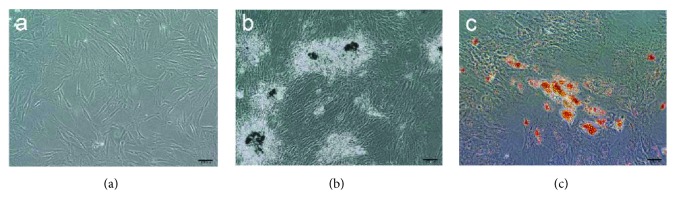
Differentiation of hASCs. (a) hASCs (p3) were observed under an inverted microscope while suspended in normal complete medium and were found to be spindle-shaped. (b) After incubating in osteogenesis medium for 3 weeks, the cells were subjected to von Kossa staining, which demonstrated the presence of calcium deposits (black). (c) Oil Red O staining confirmed that lipid droplets (orange) had formed after the cells were incubated in adipogenic differentiation medium for 2 weeks. Scale bar = 100 *μ*m. hASCs: human adipose-derived stem cells.

**Figure 2:**
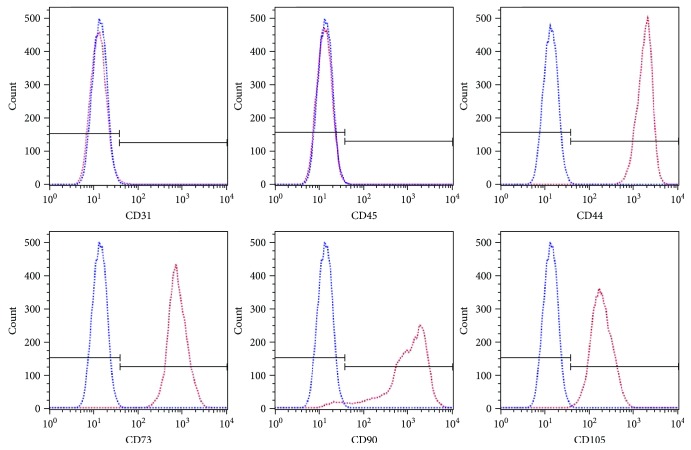
CD marker expression in hASCs was analyzed by flow cytometry. hASCs were negative (<2%) for CD31 and CD45 expression and positive (>90%) for CD44, CD73, CD90, and CD105 expression. The blue lines in each histogram indicate staining with the appropriate isotype control antibody. CD: cluster of differentiation; hASCs: human adipose-derived stem cells.

Cells at passage 3 were used in our study. The hASCs were spindle-shaped and were adherent to the culture dish, as demonstrated by inverted microscopy (Figure 1(a)). The cells were able to differentiate into cells of adipogenic and osteogenic lineages in specific induction media. von Kossa staining revealed that cells incubated in osteogenesis medium for 3 weeks exhibited calcium deposition, a phenomenon that may reflect hASC osteogenesis (Figure 1(b)), and Oil Red O staining confirmed that cells incubated in AM for 2 weeks displayed lipid droplets, indicating that the cells had acquired adipogenic properties (Figure 1(c)). In addition, flow cytometry showed that these cells were positive (>90%) for stromal markers, such as CD44, CD73, CD90, and CD105, and negative (<2%) for hematopoietic markers, such as CD31 and CD45 (Figure 2). These results were consistent with the International Federation for Adipose Therapeutics (IFATS) statement on hASC phenotypes [12].

### 3.2. Impact of the Inflammatory Environment on the miRNA Expression Profile in hASCs

**Figure 3:**
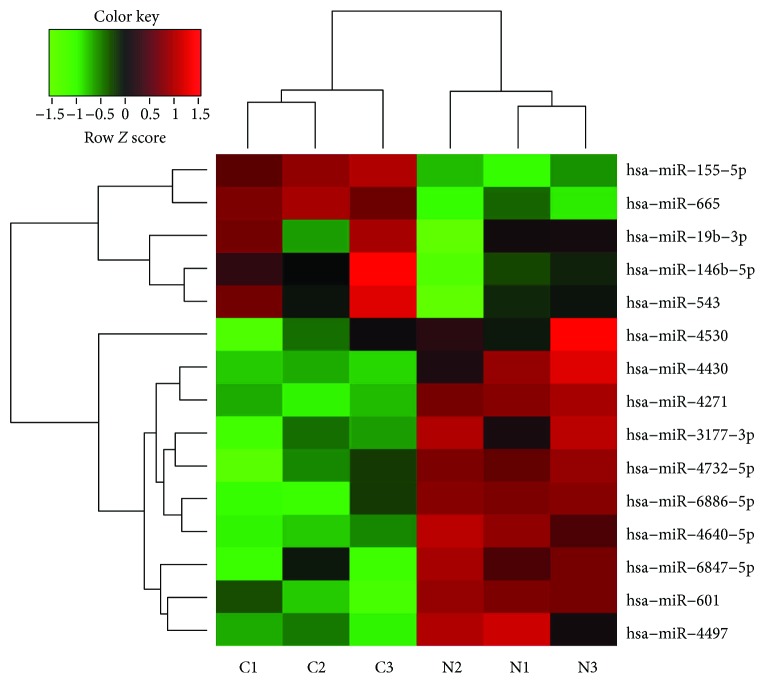
Heat map displaying the miRNAs that were differentially expressed between the normal (N) and cytokine groups (C) (*n* = 3 in each group). The heat map was drawn using Transcriptome Analysis Console software. The columns include samples from the two groups. The rows include the miRNAs that were differentially expressed between the two groups. The colors indicate miRNA expression, which increased from a relatively low (green) level to a relatively high (red) level. miRNA: microRNA.

To determine which miRNAs are differentially expressed between hASCs stimulated by an inflammatory environment and normal cells, we performed miRNA array analysis. hASCs from 3 independent donors were cultured either in normal medium (normal group) or in medium with a series of cytokines (cytokine group). RNA then was extracted from cells in each group and used for comparative analysis. The microarray analysis revealed that miRNA expression levels differed significantly between the normal and cytokine groups. A total of 6631 miRNAs were detected in the hASCs of the two groups. Among those miRNAs, 15 were differently expressed between the two groups with a |log2(fold change)| > 1 and a *p* < 0.05. Five (miR-155-5p, 665, 19b-3p, 146b-5p, and 543) of the 15 miRNAs were upregulated, and the other 10 miRNAs (miR-4530, 4430, 4271, 3177-3p, 4732-5p, 6886-5p, 4640-5p, 6847-5p, 601, and 4497) were downregulated in the cytokine group compared with the normal group. Further details regarding these miRNAs are shown in Table 1. The 15 differently expressed miRNAs are also illustrated in a heat map (Figure 3).

### 3.3. miRNA Expression Validation

**Figure 4:**
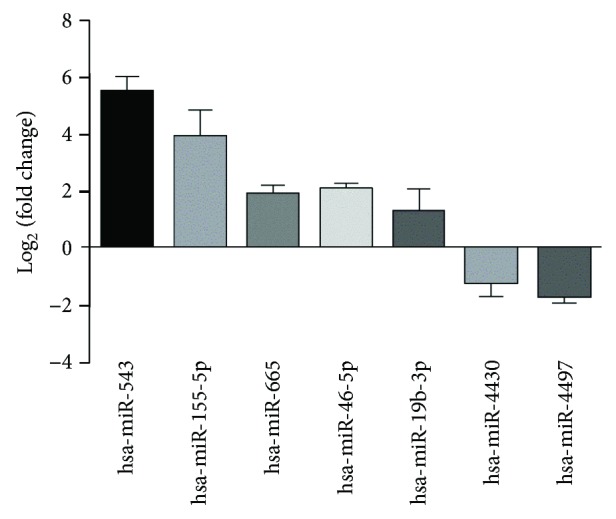
RT-PCR confirmed that 7 of the 15 miRNAs identified by the microarray displayed a fold change in their expression >2. Five miRNAs were upregulated, and 2 miRNAs were downregulated in the cytokine group compared with the control group when the cells were exposed to inflammation. RT-PCR: real-time polymerase chain reaction.

We then used RT-PCR to validate the expression levels of the 15 miRNAs that were differentially expressed between the normal and cytokine groups. Instead of adding a poly A tail to the miRNAs by reverse transcription, we used a stem-loop RT primer to improve specificity. The PCR results confirmed that 7 of the 15 miRNAs were differentially expressed between the two groups (|log2(fold change)| > 1 and *p* < 0.05). Interestingly, all 5 of the upregulated miRNAs mentioned above were successfully validated by PCR, while only 2 (miR-4430 and 4497) of the 10 downregulated miRNAs mentioned above were successfully validated (Figure 4).

### 3.4. Predicted miRNA Target Genes and Pathways

**Figure 5:**
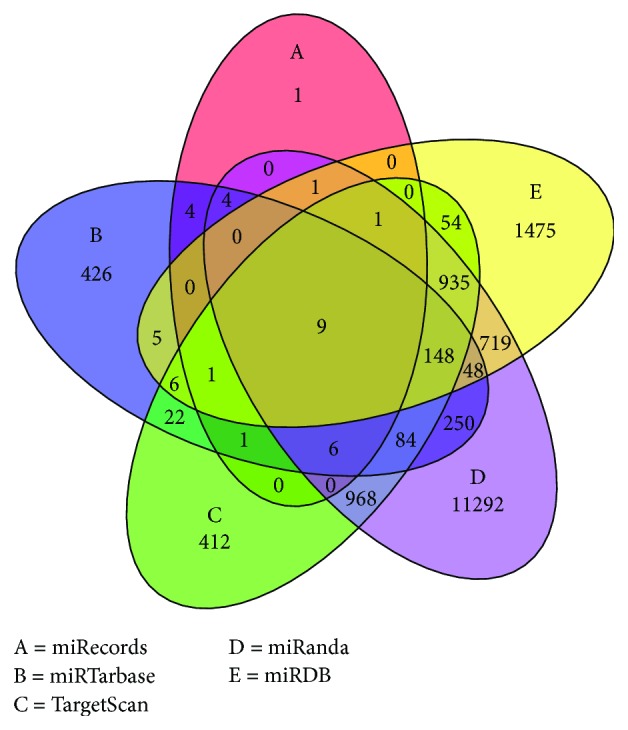
Venn diagram displaying the predicted target genes of the differently expressed miRNAs. The targets were obtained from the following 5 databases: miRecords (red), miRTarBase (blue), TargetScan (green), miRanda (purple), and miRDB (yellow). The overlaps indicate genes that were predicted by two or more databases.

The target genes of the 7 differently expressed miRNAs were predicted using 5 online databases. A total of 16,872 genes were obtained from those databases. Twenty-eight target genes were obtained from miRecords, 3402 target genes were obtained from miRTarBase, 2647 target genes were obtained from TargetScan, 14,465 target genes were obtained from miRanda, and 3402 target genes were obtained from miRDB (Figure 5). We then excluded 13,606 genes that were predicted by only one database; thus, 3266 genes were included in subsequent analyses.

**Figure 6:**
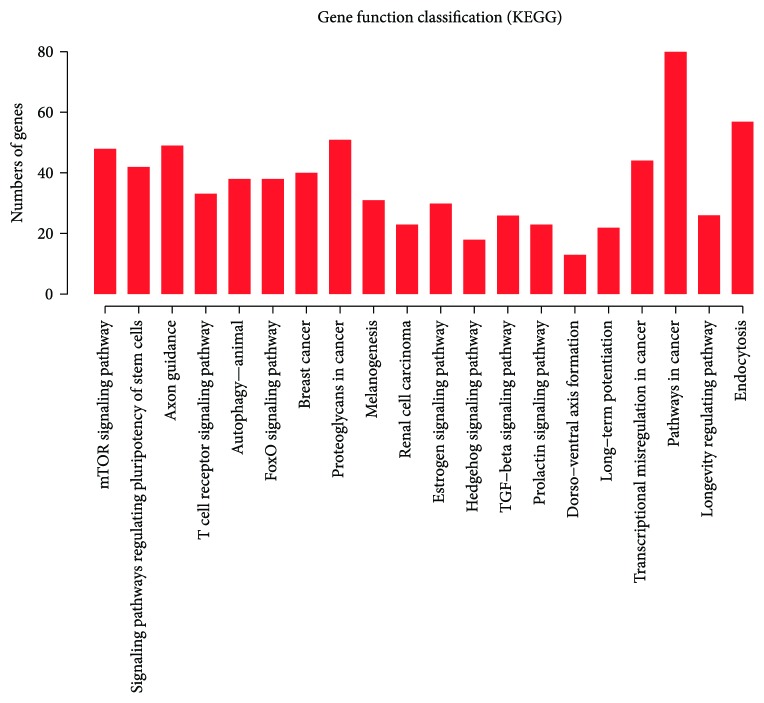
Twenty most significant related pathways, as determined by KEGG analysis. The ordinate indicates the number of predicted genes enriched in individual pathways.

Many related pathways were identified by KEGG analysis. The 20 most significant pathways are displayed in a bar-plot diagram (Figure 6). This analysis gave us an overview of all the target gene pathways that are affected by the miRNAs. We then used a miRNA pathway analysis web server (DIANA-miRPath v3.0) to identify the pathways controlled by the target genes of individual miRNAs. The inflammation-related pathways targeted by 7 differentially expressed miRNAs are summarized in Table 2. No pathways were found to be targeted by miR-4430, -4497, or -665.

## 4. Discussion

MSCs, whose existence was proposed by Caplan in 1991 [13], were identified as “colony-forming unit fibroblasts” early in the 1960s. The “stemness” of MSCs inspired scientists to apply those cells in the field of tissue regeneration, and increasing evidence provided by clinical studies has shown that MSCs are effective in tissue repair. Understanding the mechanisms by which MSCs exert their therapeutic effects will guide their application in clinical therapy. MSCs have traditionally been thought to exert their therapeutic effects by cell replacement. However, many studies have shown that successful MSC therapies are not associated with cell engraftments, indicating that other mechanisms may be involved in the effects of MSCs [14–16]. In 2004, GvHD was successfully treated in a 9-year-old child using MSC-based therapy [17]. Since that time, many clinical studies using MSC treatments [1, 18–20] have led to improvements in the clinical outcomes of patients with autoimmune diseases, findings indicating that these cells have immunoregulatory properties. However, how MSCs respond to inflammatory cytokines and regulate immunity remain unknown.

miRNAs have been shown to play an essential role in various cellular processes by repressing the translation of target mRNAs. Abnormal miRNA expression profiles may be related to different human diseases, including inflammatory diseases, indicating that some small noncoding RNAs have immunoregulatory functions [3]. We hypothesized that cross-talk between MSCs and inflammatory stimuli occurs through regulated miRNA expression.

hASCs, which are isolated and expanded from adipose tissue, are a promising type of MSC. In this study, we used a set of inflammatory cytokines to stimulate hASCs and then explored the subsequent changes in the miRNA profile of the cells using microarray analysis. We confirmed that 7 miRNAs were significantly differentially expressed between the cytokine and normal groups. The following 5 miRNAs were upregulated: miR-155-5p, 665, 19b-3p, 146b-5p, and 543. The following 2 miRNAs were downregulated: miR-4430 and -4497.

MiR-543 was the miRNA whose expression was upregulated most significantly in the cytokine group compared with the normal group. Specifically, miR-543 expression levels differed by more than 40-fold between the two groups. In one study, miR-543 was shown to regulate MSC aging by decreasing AIMP3/p18 expression [21]. The other upregulated miRNAs are all known to be related to inflammatory diseases. MiR-155 and miR-19b-3p have been demonstrated to modulate Japanese encephalitis virus- (JEV-) mediated inflammation [22, 23]. MiR-155 has also been reported to reduce the immunosuppressive capacity of MSCs [24] and is upregulated in foreskin-derived MSCs following priming by inflammation [25]. MiR-665, 146b-5p, and 155-5p were all upregulated in the inflamed colon of patients with ulcerative colitis [26], and miR-146b-5p has also been shown to be upregulated in the rheumatoid arthritis synovial tissue [27] and to be involved in the immunosuppressive functions of human retinal pigment epithelial cells [28].

Those studies and our results serve as strong evidence showing that miRNAs play a pivotal role in the immunoregulatory properties of hASCs. To understand the mechanisms by which these miRNAs regulate the immune response further, we predicted the target genes of the miRNAs and performed pathway analysis. Unsurprisingly, we identified several immune-related pathways, such as the T cell receptor signaling pathway, the FoxO signaling pathway, and the TGF-*β* signaling pathway. We also analyzed the pathways targeted by individual miRNAs. MiR-543, 155-5p, 146b-5p, and 19b-3p all target immune-related target pathways. We also noticed that miR-155-5p and 146b-5p target the NF-*κ*B signaling pathway, a finding consistent with those of previous studies [4].

Autologous hASCs can be easily obtained with minimal pain or side effects. Therefore, the clinical application of hASCs is very promising, and the therapeutic effects of these cells in certain diseases have already been reported [1]. Elucidating the mechanism by which hASCs exert their immunoregulatory effects may improve their therapeutic efficacy. The differently expressed miRNAs identified in this study may serve as therapeutic targets, and overexpressing miRNAs with anti-immunity functions may enhance the immunosuppressive properties of hASCs. However, the specific functions of these miRNAs in hASCs in vitro and in vivo still need further elucidation.

## 5. Conclusion

We first found that the miRNA expression profile of hASCs changes significantly under inflammatory stimulation. Five miRNAs were upregulated, and 2 miRNAs were downregulated in the cytokine group compared with the control group. These differently expressed miRNAs may participate in several immune-related pathways in the immune response and may thus serve as targets through which the immunoregulatory functions of hASCs can be enhanced.

## Figures and Tables

**Table 1:** miRNAs that were differentially expressed between the cytokine and normal groups.

miRNA	Fold change (cytokine versus normal group)	*p* value
hsa-miR-665	4.64	0.01
hsa-miR-146b-5p	4.13	0.04
hsa-miR-155-5p	4.00	<0.01
hsa-miR-19b-3p	2.10	0.01
hsa-miR-543	2.02	0.01
hsa-miR-4430	0.49	0.03
hsa-mir-4530	0.47	0.01
hsa-miR-6847-5p	0.44	0.02
hsa-miR-4497	0.42	0.02
hsa-miR-4732-5p	0.40	0.02
hsa-miR-4640-5p	0.34	0.02
hsa-miR-6886-5p	0.33	0.02
hsa-miR-3177-3p	0.32	<0.01
hsa-miR-601	0.27	0.02
hsa-miR-4271	0.18	<0.01

The microarray identified 15 miRNAs with a *p* < 0.01 and a |log2(fold change)| >1 in the two groups.

**Table 2:** Immune-related pathways predicted to be targeted by individual miRNAs.

miRNA	ID	Description	*p* value	Gene count
hsa-miR-543	hsa04350	TGF-beta signaling pathway	0.0004	17
hsa-miR-543	hsa04068	FoxO signaling pathway	0.0024	26
hsa-miR-543	hsa04917	Prolactin signaling pathway	0.0464	14
hsa-miR-155-5p	hsa04068	FoxO signaling pathway	0.0003	26
hsa-miR-155-5p	hsa04350	TGF-beta signaling pathway	0.0003	11
hsa-miR-155-5p	hsa04064	NF-kappa B signaling pathway	0.0026	16
hsa-miR-146b-5p	hsa04064	NF-kappa B signaling pathway	0.0298	7
hsa-miR-146b-5p	hsa04620	Toll-like receptor signaling pathway	0.0298	10
hsa-miR-146b-5p	hsa04668	TNF signaling pathway	0.0480	10
hsa-miR-19b-3p	hsa04917	Prolactin signaling pathway	0.0002	18
hsa-miR-19b-3p	hsa04068	FoxO signaling pathway	0.0002	30
hsa-miR-19b-3p	hsa04350	TGF-beta signaling pathway	0.0417	12
